# An Empirical Study on Drivers’ Willingness to Use Automatic Features of Intelligent Vehicles: A Psychological Empowerment Perspective

**DOI:** 10.3389/fpsyg.2021.794845

**Published:** 2021-12-16

**Authors:** Ting Li, Sumeet Gupta, Hong Zhou

**Affiliations:** ^1^School of Management, Huazhong University of Science & Technology, Wuhan, China; ^2^Department of Operations and Systems, Indian Institute of Management Raipur, Raipur, India

**Keywords:** intelligent vehicles, automatic features, perceived cognitive empowerment, perceived emotional empowerment, perceived behavioral empowerment

## Abstract

With the advancement in AI and related technologies, we are witnessing more remarkable use of intelligent vehicles. Intelligent vehicles use smart automatic features that make travel happier, safer, and efficient. However, not many studies examine their adoption or the influence of intelligent vehicles on user behavior. In this study, we specifically examine how intelligent vehicles’ sensing and acting abilities drive their adoption from the lens of psychological empowerment theory. We identify three dimensions of users’ perceived empowerment (perceived cognitive empowerment, perceived emotional empowerment, and perceived behavioral empowerment). Based on this theory, we argue that product features (sensing and acting in intelligent vehicles) empower users to use the product. Our proposed model is validated by an online survey of 312 car owners who are familiar with driving conditions, the results of this study reveal that driver’s perceived empowerment is vital for using automatic features of intelligent vehicles. Theoretically, this study combines the concept of empowerment with the intelligent-driving scenario and reasonably explains the mechanism of the intelligence of vehicles on users’ behavior intention.

## Introduction

Intelligent vehicles are one of the latest inventions that stem from technological advancements in artificial intelligence, big data, cloud computing, the Internet of things, and 5G. Although in the nascent stages of development, the market is fertile for its growth. The shipments of intelligent vehicles have grown substantially during the last few years (IDC’s Global Automobile Forecast Report, 2020–2024). In China, the market volume of intelligent vehicles has already crossed 13 million by 2020, as is evident from the sales data of the top three Chinese EV startups (NIO, Li Auto, and XPENG). McKinsey (McKinsey China, 2018) reports that China will likely become the largest intelligent driving market globally and create a $500 billion market by 2030. Policies regarding its adoption are also pretty favorable in China. The National Development and Reform Commission estimates that the penetration rate of intelligent vehicles will reach 82% by 2025, with the number reaching 28 million (Jiemian, 2020). Consequently, several startups, including tech giants, such as Huawei, Apple, and Alibaba, have popped up to grab this market share. Xiaomi also announced an investment of 10 billion yuan in this industry, and Baidu took the lead in launching a free self-driving taxi service in Beijing in October 2020.

Previous studies (Contreras-castillo, 2020) define intelligent vehicles as those that can sense their surroundings and act accordingly (such as plan the route and safely maneuver through it). The sense-act paradigm has been drawn from robotics as robots first sense and then act accordingly (Garattoni and Birattari, 2018). Intelligent vehicles predict obstacles using lidar, ultrasonic, and infrared and adjust their speed according to the road conditions using the adaptive cruise control (ACC) feature. Other intelligent features, such as advanced driver assistance systems (ADAS), also empower drivers through dynamic path planning, path reminding, automated driving, and automatic parking.

Considering the favorable market of intelligent vehicles, we wish to examine the factors that enhance their adoption. Previous studies have examined their adoption using the cost–benefit perspective (Liu and Xu 2020) or seeking individual and expert opinions about their adoption (Kim et al., 2019). While the cost–benefit paradigm has been successfully used for examining technology adoption, the adoption of intelligent vehicles is closely related to their ability to sense and act accurately. While intelligent vehicles make life easy, adoption is difficult as inaccurate actions may endanger the driver’s life. Most studies, however, have not considered this perspective in examining their adoption. Previous studies (e.g., Wang et al., 2019) make a strong case for their adoption using psychological factors. Studies on the adoption of smart services also consider functional, psychological, and individual aspects of products (Mani and Chouk, 2018) and not just the cost–benefit perspective. We, therefore, borrow from the empowerment theory to examine their adoption. We argue that drivers feel empowered to drive safely and better due to intelligent vehicles’ sensing and acting abilities (Maas et al., 2018). Previous studies have applied this theory to understand how technology empowers users (Li et al., 2017) and influences their behavior (Zha et al., 2020) and to study patient empowerment in healthcare (Ouschan et al., 2000) and interpersonal relationships (Beer et al., 2014).

Therefore, in this study, we examine the adoption of intelligent vehicles from the lens of psychological empowerment based on their capability to sense and act. The specific research questions we seek to explore are as: *How do sensing-acting abilities of intelligent vehicles psychologically empower users to consider their adoption*? *How does the sensing-acting paradigm influence driver’s adoption of intelligent vehicles*? This paper also considers the three dimensions of perceived empowerment to fill this crucial research gap in the intelligent-driving context.

The rest of the paper is organized as follows. In the second section, we review the existing literature on related research topics, including intelligent vehicles and empowerment theory. In the third section, we present the research framework and hypothesis on how the sensing-acting abilities empower users and influence users’ adoption of intelligent vehicles. In the fourth section, we present the methodology for collecting data as well as the analysis of the collected data. In the fifth section, we present the discussion and implications of our study, followed by conclusions in the sixth section.

## Literature Review and Theoretical Background

### Intelligent Vehicle Usage

Previous studies (Manfreda and Ljubi, 2021) investigated consumers’ adoption of smart products based on the TAM model. There have been several extensions and modifications in the original TAM and UTAUT model, and these modified models also explain consumers’ adoption of smart products (Mosquera et al., 2018). Among the IS studies, Baccarella et al. (2020) explored users’ behavioral intention based on the TAM model and found that perceived usefulness and perceived ease of use significantly influence the usage intention. Similarly, Just et al. (2017) also found support for these variables when using UTAUT as the theoretical basis for examining usage intention of intelligent vehicles. Although intelligent vehicles provide drivers with comfort, relaxation, navigation, and time savings (Propagation, 2018), they also carry perceived risks. What if the intelligent vehicle is unable to judge the situation correctly? Rana et al. (2016) and Mosquera et al. (2018) argue that context-specific and individual-focused modifications are missing in these studies. Furthermore, not all moderators are justified in all contexts, and we should consider them on a case-to-case basis.

Although previous studies provide evidence of people’s intention to use intelligent vehicles, they do not consider product characteristics and the psychological effect in examining the adoption of intelligent vehicles. A few previous studies (Wang et al., 2019) provide evidence that product characteristics and psychological effects are two important factors that influence the adoption of intelligent vehicles. Technology adoption theories, such as TAM and UTAUT, only consider perceived usefulness and perceived ease of use in examining usage intention. However, in the case of intelligent vehicles, their intelligence also plays an essential role in their adoption. At least a user needs to become sufficiently convinced that the intelligent vehicle would not put them in a difficult situation. As there is a risk of life, considering product characteristics is extremely important in examining their adoption compared to other smart products (Kang and Kim, 2020). Secondly, technology adoption theories do not consider the perceived empowerment that believes how intelligent vehicles empower drivers with skills and help them drive with ease.

Therefore, to promote intelligent vehicles, it is crucial to building confidence among users toward their use. Several studies (e.g., Wang et al., 2019; Chowdhury et al., 2021; Manfreda and Ljubi, 2021) have found support for the influence of attitude on the acceptance of intelligent vehicles. These studies indicate that the direct experience (such as a test ride on intelligent vehicles) induces a positive attitude in participants toward their acceptance. Other studies (e.g., Wang et al., 2019; Liu and Xu, 2020) have examined their acceptance from the perspective of innovation diffusion. These studies conclude that besides the psychological factors (awareness, expectation, trust, and concern), product characteristics (technology function and cost) also play an important role in their adoption. Considering these studies, we also consider context-specific factors in examining intelligent vehicle adoption.

We argue that intelligent vehicles empower users because of the intelligence embedded in them. They help avoid traffic jams by assisting users with critical driving tasks (such as destination path planning, automatic parking, and early warning of impending risks), thus freeing them from cognitive, psychological, and physical fatigue (Baraglia et al., 2016). Compared to traditional vehicles, they make driving more convenient by helping users carry out dynamic path planning, automatic parking, adaptive cruising, and automatic lane changing. Although intelligent vehicles could make our life easier due to their technological innovation and product quality, public concerns also exist. Hence, their marketing and commercialization are just as important (Hengstler et al., 2016). In other words, the primary obstacles to the adoption of intelligent vehicles are psychological than technical (Shariff et al., 2017). We use psychological empowerment theory to examine how intelligence empowers drivers to adopt intelligent driving.

### Psychological Empowerment Theory

Psychological empowerment theory has its origins in industrial-organizational psychology. Empowerment refers to the opportunity an individual has for autonomy, choice, responsibility, and decision making. It is a process through which people, organizations, and communities gain mastery over issues of concern to them (Zimmerman, 1995). Several studies have applied the concept of empowerment in various fields, such as psychology (Thomas and Velthouse, 1990), management (Conger and Kanungo, 1988), and medical science (Gustafson et al., 1999) at the individual, organizational, and community level. Psychological empowerment is an intrinsic task motivation reflecting a sense of self-control concerning one’s work and an active engagement with one’s work role (Seibert et al., 2011). Psychological empowerment stimulates individuals’ enthusiasm for performing a task or for taking a decision. Early empowerment research concentrated on the characteristics of users and institutional factors in society and organizations. However, studies have not used it in examining intelligent vehicles or smart products. Although psychological empowerment stems from how leadership in an organization promotes an environment of empowerment, products may also empower users to perform tasks they otherwise would not achieve. For example, typing was a task of specially trained typists. But after the advent of computers and word processing software, individuals became empowered to do so with little training in typing. Following previous literature (Seidmann and Sundararajan, 1997), we suggest that empowerment means giving the power to do something one would not otherwise be able to do, thus encouraging participants in more decisions and activities.

A few IS studies have examined the relationship between technology and users based on the empowerment theory, and they consider technology as empowering users in different situations. For example, Li et al. (2017) argue that psychological empowerment can explain the effect of virtual counselor status on the psychological rescue of emergency responders in a psychological self-help system. The proposed a low-cost and widely deployable strategy for empowering emergency rescuers using an intelligent mobile psychological self-help tool. Their experiment confirms that the device influenced a user’s cognitive and emotional routes that led to positive empowerment outcomes. The results also show users’ needs will determine the routes of empowerment. The peer identity empowers users through emotional routes, and the expert identity empowers them through cognitive routes.

Similarly, Li et al. (2021) proposed using the empowerment framework to examine the mechanism of the gamified psychological autonomous system on a users’ psychology. Specifically, they explored the value of a user’s affective experience mirroring and examined the empowerment effect of meaningful gamification in a self-help system that aided people under work stress. From the outcomes of empowerment,users generally perceived the visual impact metaphor as arousing more positive feelings, relieving their stress, and enhancing their happiness. Li and Gregor (2011) use empowerment theory to describe how the government website acts as a critical consulting tool to improve citizens’ sense of power. The findings indicate that citizens feel empowered when e-government websites are transparent and feel a sense of control when online advisory tools are present on the site. Hence, we propose that user empowerment improves one’s confidence, self-efficacy, and control, ultimately reflected in their behavior.

### Perceived Empowerment

Previous studies have focused on users’ empowerment as a predictor of technology adoption (Stodder, 2015; Vilela et al., 2015). Deibel (2011) defined user empowerment in organizations as the ability and behavior of users to freely plan, decide and perform work-related tasks in the way they deem best. Intelligent products empower users by improving their capacity and efficiency during driving (Speed et al., 2013). Daradkeh (2019) showed that empowerment could stimulate users to perform better reporting and analysis with more resources and freedom to work. He also reported that empowerment promotes users to try new technologies and new procedures to perform tasks better. The concept of empowerment has been successfully examined in several consumer and IS studies. Kozinets et al. (2004) argue that the internet empowers users with increased information, greater choice, and control. Similarly, Hu and Krishen (2019) argue that consumers feel cognitively and emotionally empowered to make purchase decisions because of the availability of online reviews. These studies suggest that psychological empowerment includes the cognitive, emotional, and behavioral components. Several previous studies have confirmed the same (e.g., Speer and Peterson, 2000; Christens, 2012). Christens (2012) expanded the concept of psychological empowerment by theorizing it as a construct with emotional, behavioral, and cognitive dimensions. Other studies, such as Li et al. (2017) and Hu and Krishen (2019), also demonstrate that users feel empowered through cognitive and emotional routes.

We summarize these dimensions in Table 1. In summary, we argue that cognitive, emotional, and behavioral components influence consumer decisions. Therefore, based on previous studies, we divide perceived empowerment into three dimensions (cognitive, emotional, and behavioral) to examine the empowerment process in detail. The cognitive dimension enhances our understanding of the surrounding environment and about intelligent functions in the vehicle. The emotional dimension examines how intelligent vehicles make drivers more confident in the driving process. The behavioral dimension examines how intelligent vehicles help perform operations more skillfully.

**Table 1:** Summary of the dimensions of psychological empowerment.

Reference	Dimensions of empowerment	Definition
Speer and Peterson, 2000	Cognitive empowerment	Critical awareness and understanding of community functioning.
	Emotional empowerment	Feelings about one’s competence or ability to effect change in the community.
	Behavioral empowerment	Participatory activities focused on social change in community contexts.
Christens, 2012	Cognitive empowerment	Skills and critical understandings are necessary for exerting sociopolitical influence.
	Emotional empowerment	Self-perceptions of one’s competence in exerting influence in the sociopolitical domain.
	Behavioral empowerment	Directly to the actions taken to exert influence.
Li et al., 2017; Hu and Krishen, 2019	Cognitive empowerment	Imparting knowledge or information as well as providing users with autonomy and delivering freedom through choices.
	Emotional empowerment	Affiliation, support, or positive effect can be linked to social interactions to facilitate communication.
Zimmerman, 1995; Speer, 2000	Intrapersonal empowerment	Think of themselves in terms of the exercise of control, motivation to control, and perceived self-efficacy in a specific context.
	Interactional empowerment	Understand the social environment around them to build a critical understanding of the forces that shape the social environment around them.
	Behavioral empowerment	Take actions to produce desired social changes.

## Research Model and Hypotheses

**Figure 1:**
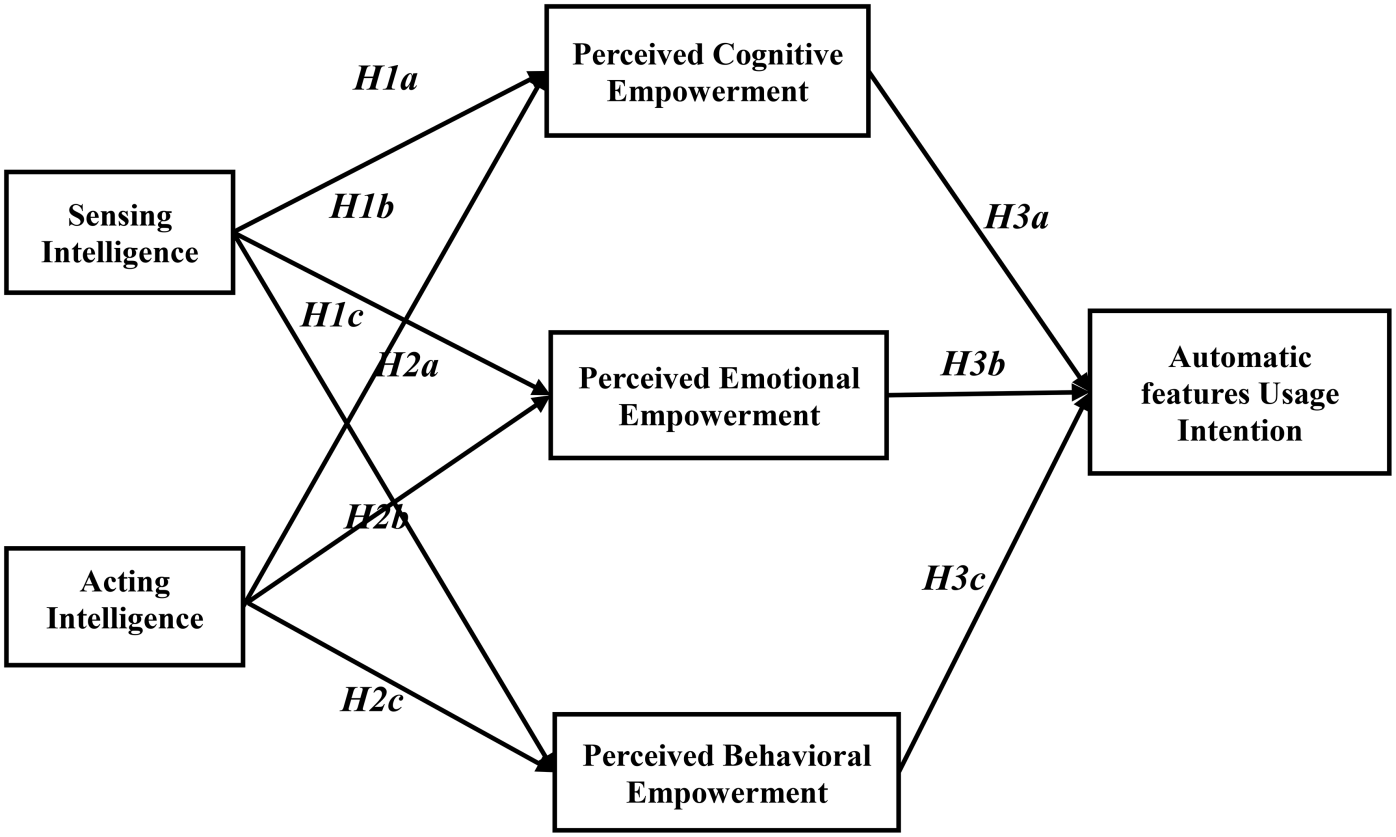
Research model and hypotheses.

In this study, we categorize the intelligence of the vehicle into sensing intelligence and acting intelligence. We consider these as the predictors of empowerment enablers. In line with the empowerment theory, we investigate how the intelligence of vehicles affects users’ usage intention through three essential dimensions of empowerment (perceived cognitive empowerment, emotional empowerment, and behavioral empowerment). A few studies mentioned that information technologies, such as the internet, increase users’ power (Amichai-Hamburger et al., 2008). Studies (e.g., Kim et al., 2019) reveal that intelligent vehicles bring several benefits to consumers and society. However, to examine their usage intention, we use a broader framework that includes the intelligent characteristics of products and users’ psychological factors. Figure 1 presents this framework as a research model depicting the proposed hypothesis.

### The Effect of Intelligence on Perceived Empowerment in Intelligent Vehicles

As discussed earlier, empowerment enables people to do things that they could not perform earlier (Harrison and Waite, 2015). Staples (1990) consider empowerment as an improved ability in an individual to interact with the world. Intelligent vehicles may empower users in several ways through their sensing and acting abilities (Amichai-Hamburger, 2008). They predict obstacles using lidar, ultrasonic, and infrared and adjust their speed according to the road conditions using the adaptive cruise control (ACC) feature. Other intelligent features like the advanced driver assistance system (ADAS) empower drivers through dynamic path planning, path reminding, automated driving, and automatic parking.

#### The Effect of Sensing Intelligence on Perceived Empowerment

Xu et al. (2020) describe the sensing intelligence of the vehicles as the perception of things happening around them. Barakova et al. (2018) proposed that sensing of intelligent vehicles includes how the vehicle perceives the environment. The sensing intelligence in our study refers to the *ability of intelligent vehicles to detect the surrounding environment, perceive information, collect information in time, store, and obtain information*. Galanis et al. (2019) argue that sensing intelligence could increase a driver’s cognitive awareness about any possible danger in the observed environment. In other words, it improves the coverage of the observation area and provides better knowledge and perception of the driving environment. The intelligent vehicles thus empower drivers by monitoring the surroundings and alerts them of impending road conditions, thus releasing them of fatigue due to high concentration during driving. Hence, we hypothesize as:

*H1a: Sensing will have a positive influence on perceived cognitive empowerment.*

Pagallo (2013) highlights that intelligent vehicles increase users’ confidence and positive emotions, especially in complex driving conditions, by providing timely reminders to make the driving experience safer, comfortable, and efficient, and thus help users become familiar with the surroundings and drive with ease. Moreover, sensing allows drivers to communicate with the environment as vehicles transmit helpful information through sensors, thus increasing their sense of control. In general, sensing could help empower users with emotional value to some extent. Hence, we hypothesize as:

*H1b: Sensing will have a positive influence on perceived emotional empowerment.*

Additionally, Hu et al. (2021) suggest that sensing for robots and other smart products usually refers to the extent to which intelligent personal assistants (IPAs) can sense the environment to perform assigned tasks. Better perception reduces the driver’s physical burden and stress by creating a more accurate perception of the environment, thus reducing unnecessary hassles by simplifying the driving process. According to Wang et al. (2020), perception is a crucial aspect in the automatic driving systems, and the driving behavior is closely related to the perception of the environment. Hence, we hypothesize as:

*H1c: Sensing will have a positive influence on perceived behavioral empowerment.*

#### The Effect of Acting Intelligence on Perceived Empowerment

Barakova et al. (2018) proposed that acting intelligence in intelligent vehicles refers to the actions performed by the vehicle as a response to sensing. Similarly, Xu et al. (2020) refer to the acting function of intelligent vehicles as “actions taken to maximize their utility.” This study refers to acting intelligence as the response of intelligent vehicles and assistive driving activities based on the perceived information. When applied to the intelligent driving context, the acting intelligence of intelligent vehicles helps users by controlling the speed and direction of the vehicle, which reduces errors arising from fatigue and medical emergencies. The cruise control systems of intelligent vehicles control the speed through the steering wheel to keep a safe distance from the car in front or even apply breaks during an emergency. The actions of intelligent vehicles could help users become more familiar with the road conditions and relieve mental stress due to driving. Moreover, when drivers share the assigned tasks with intelligent vehicles, their cognitive pressure gets reduced due to alleviating intense and focused attention. Hence, we hypothesize as:

*H2a: Acting will have a positive influence on perceived cognitive empowerment.*

Accompanying the user as an intelligent assistant can not only alleviate loneliness but also make driving more enjoyable. Intelligent vehicles help users perform tasks they would otherwise need to do independently, sometimes playing specific songs, opening windows, and air conditioners, or adjusting seats to a proper position, creating a comfortable, and pleasant driving experience. On the other hand, when faced with complex conditions, intelligent vehicles can assist in a timely, professional, and skilled manner to make drivers feel more confident while driving. In particular, the automatic parking system helps users find empty parking spaces and assist them in parking, thus reducing their anxiety and increasing confidence and positive emotions in them. Hence, we hypothesize as:

*H2b: Acting will have a positive influence on perceived emotional empowerment.*

The action of intelligent vehicles helps users drive better and thus reduces their burden. In addition, it keeps steering control and active acceleration or deceleration, and longitudinal control to avoid obstacles. At the same time, it can also carry out parallel lane assistance, lane departure warning, lane-keeping assistance, and other side control functions. Based on these features mentioned, the functional skills and activities of the driver get enhanced, thus ensuring safe driving. Hence, we hypothesize as:

*H2c: Acting will have a positive influence on perceived behavioral empowerment.*

### The Effect of Perceived Empowerment on Usage Intentions

Scholars note the positive results of perceived empowerment. Scholars (e.g., Conger and Kanungo, 1988; Kirkman, 1999; Chamberlin et al., 2018) argue that high perceived empowerment is positively correlated with team performance. Furthermore, some authors (e.g., Holosko et al., 2001) argue that workers display positive behavior when the organization empowers them. Fernandez (2013) highlights that perceived empowerment influences performance directly as well as job satisfaction and innovation. Other studies (e.g., Joo and Shim, 2010; Humborstad and Perry, 2011) highlight the importance of perceived empowerment on organizational commitment, quality of service, job satisfaction, and innovative effectiveness and behavior.

Perceived empowerment refers to giving power or letting people do something they cannot do by themselves and encouraging them to participate more in decisions and activities (Maas et al., 2018). Previous IS studies (e.g., Li et al., 2017, 2021) used the empowerment theory to explain the relationship between information systems and users. These studies conclude that various applications use information technology as an empowerment enabler. For instance, Li et al. (2017) first interpret that the self-help systems could empower rescuers with control and power, releasing them from highly stressful careers. The system influences user’s cognitive and emotional routes, which empower them and lead to positive outcomes. Similarly, Li et al. (2021) argue that a psychological self-help system arouses users’ feelings of enjoyment, empathy, trust, and usefulness and empowers them with greater mastery and control over themselves. Thus, these studies emphasize that a virtual system facilitates users’ empowerment by gaining their appreciation, establishing a deeper connection with them, and eventually leading to beneficial outcomes.

In summary, empowerment aims to improve a driver’s sense of self-efficacy, control, and confidence further reflected in their behavior and activities. We extend these arguments to the context of intelligent vehicles. Perceived empowerment refers to enabling people to drive intelligent vehicles more safely. Moreover, intelligent vehicles help users do better with (lesser effort than earlier). As mentioned earlier (Li et al., 2021), users’ feelings of usefulness, pleasure, empathy, and trust are evoked by perceived empowerment.

A few studies point out that psychological empowerment improves users’ affective and normative commitment, which illustrates that perceived empowerment positively affects their behavioral intention (Nayak et al., 2018). Further, users’ perceived empowerment will increase their engagement (Füller et al., 2009). Considering these positive effects of perceived empowerment, we conclude that drivers would consider using automatic features in intelligent driving scenarios. Hence, we hypothesize as:

*H3a: Perceived cognitive empowerment will have a positive influence on users’ intention to use automatic features.*

*H3b: Perceived emotional empowerment will have a positive influence on users’ intention to use automatic features.*

*H3c: Perceived behavioral empowerment will have a positive influence on users’ intention to use automatic features.*

### The Moderating Effect of Sense of Control

Sense of Control (SoC) is a vital outcome of the empowerment process as control comes with power as an outcome of empowerment (Li et al., 2017). Rotter (1966) first proposed the concept of SoC as a Locus of control in social psychology. SoC refers to how an individual believes that they can change an outcome under various situations and actions. In addition, scholars (e.g., Zimmerman, 2000) found that perceived control reduces psychological stress. Additionally, Gatchel (1980) argues that individuals react differently to situations perceived as controllable than those seen as uncontrollable. Investigators have also found perceived control related to psychological stress and positive behaviors, which provide a basis for studying the perceived empowerment (Visher, 1986; Fleming et al., 1987).

An SoC over the intelligent vehicle is critical for users, especially during different driving environments (Marina Martinez et al., 2018). Wang et al. (2019) argue that SoC is extremely important in the evaluation of intelligent vehicles. In the context of intelligent driving, SoC refers to the extent to which users believe that their actions can change the outcome of driving. At present, the interplay of control and empowerment remains unclear, especially in the context of intelligent vehicles. Previous studies (e.g., Tennant et al., 2011; Maas et al., 2018) have examined perceived control and perceived empowerment at the individual level from a longitudinal perspective and concluded that both the level of control and empowerment remains consistent over time. Research (e.g., Li and Gregor, 2011) shows that a sense of control implies better empowering effects in the context of e-government.

In summary, SoC is a crucial variable and influences the empowerment process. Several reports on safety risks in intelligent vehicles reveal that the loss of control is a reason for accidents. Hence, control over the vehicles may be a prerequisite to ensure users’ trust and safety. Therefore, a sense of control may improve user’s intentions to use automatic features. Hence, we hypothesize as:

*H4a: SoC will enhance the relationship between perceived cognitive empowerment and usage intention.*

*H4b: SoC will enhance the relationship between perceived emotional empowerment and usage intention.*

*H4c: SoC will enhance the relationship between perceived behavioral empowerment and usage intention.*

## Materials and Methods

We collected the data for testing our research model using an online survey. We developed a questionnaire based on the existing literature to examine users’ attitudes toward automatic features of intelligent vehicles.

### Measurement Development

We adapted the measures from previous studies to the context of this study. We tested them on a five-point Likert scale. We adopted measures for sensing intelligence and acting intelligence from Hu et al. (2021). We adopted measures for cognitive empowerment from Christens (2012) and Aleksander (2017). We adopted emotional and behavioral empowerment measures from Christens (2012) and Speer and Peterson (2000). We adopted measures for automatic features usage intention from Komiak and Benbasat (2006), Qiu and Benbasat (2008). We measured all latent model variables on a five-point Likert scale ranging from “strongly disagree” (1) to “strongly agree” (5). Appendix A presents the operational definitions of the constructs together with their subdimensions and sources.

### Pretest of Automatic Features Usage Intention

We first invited five experts and 15 scholars in the field of artificial intelligence to pretest the questionnaire. Based on their feedback on the face and content validity, we revised the questionnaire. Then, we conducted a pilot test to examine the quality of items. We invited subjects randomly by publishing the questionnaire to Wenjuanxing,^1^ a professional online questionnaire platform, as well as Amazon’s Mechanical Turk (m-Turk). We analyzed the responses after we had collected 100 usable responses. The results revealed that all Cronbach’s alphas were above 0.7, implying that the questionnaire was proper. We made minor revisions to the questionnaire based on the comments given by respondents in the pilot study.

### Data Collection

We collected the data by publishing the survey on Wenjuanxing. We offered monetary incentives to respondents for participating in the survey. Since the survey was regarding intelligent vehicles, we presented the respondents with use cases to make them aware of the automatic features of sensing and acting. To test their understanding of intelligent vehicles, we asked a few questions. If the respondents answered them correctly, they could proceed to complete the questionnaire further. We used non-probabilistic sampling. We also collected information on the socio-demographic characteristics of the respondents. We recruited participants beginning July 2021, and the process of data collection lasted 2 weeks. We obtained a total of 350 responses. After processing the data (treatment of missing values, the analysis of outliers, and normality tests), we were left with 312 responses. Table 2 presents the summary of the characteristics of respondents.

**Table 2:** Demographics of the research sample.

Variable	Category	N	Percentage (%)
Gender	Male	215	68.9
	Female	97	31.1
Age	18–25	23	7.4
	26–35	213	68.3
	36–45	69	22.1
	>45	7	2.2
Education	High school or lower	6	1.9
	College degree	43	13.8
	Bachelor’s degree	236	75.6
	master’s degree or higher	27	8.7
Marriage	Unmarried	57	18.3
	Married and childless	26	8.3
	Married with children	229	73.4
Annual income/yuan	<100,000	47	15.1
	100,000–200,000	147	47.1
	200,000–300,000	83	26.6
	300,000–500,000	28	9.0
	>500,000	7	2.2
Commuting distance	<5Km	26	8.3
	5–10Km	115	36.9
	11–15Km	93	29.8
	16–20Km	46	14.7
	21–25Km	19	6.1
	>25Km	13	4.2

The sample of 312 consisted of 68.9% males and 31.1% females. This is consistent with the ratio released by the Intelligent vehicle industry research report of 2020 (Sina, 2020). Thus, our sample represents the target group of intelligent vehicles user. Our sample matched that described in this report – young, highly educated, and high disposable income. Almost 70% of respondents in our sample were between 26 and 35 years old, nearly 85% of respondents had a bachelor’s degree or above, and 75% of respondents earned 100,000 yuan or more per year.

### Data Analysis and Results

As the responses were collected using a cross-sectional survey and the measures were self-reported, we tested for the possibility of common method bias (CMB). First, we carried out Harman’s single-factor test. The results reveal three factors, out of which the first factor contributes about 25.3%. Since this does not account for most of the covariance in the variable, CMB is not a threat in our study. Secondly, we also evaluated the common method variance using the common method factor to eliminate CMB’s risk further. The results were generated by Smart-PLS 3.0. We constituted a single variable for all measurement items and regarded each indicator as a separate variable. We found that the explanatory variance rate of all latent variables for each indicator was much higher than that of the marked variable. Thus, CMB is not a problem in our study.

#### Measurement Model Test

We used structural equation modeling (SEM) to test both the measurement and structural model using Smart-PLS 3.0 software. Structural equation modeling (SEM) is suitable for the model proposed in this paper. Table 3 shows the results of the measurement model test. We note that all standardized item loadings for latent variables are above 0.8, and t-values are significant at a 99% confidence interval. In addition, Cronbach’s alphas for factors identified in the proposed model are above 0.7, CR ranges from 0.831 to 0.920, and AVE for each factor ranges from 0.551 to 0.776. Thus, the measurement model of study is reliable (Nunnally, 1975). Further, AVE is greater than 0.5 for all latent variables, indicating that the underlying latent variables explain more than half of the variance in the indicators (Yi, 1988).

**Table 3:** Results of confirmatory factor analysis.

Construct	Items	T-value	Loading	Cronbach’s Alpha	Composite reliability	Average variance extracted
Sensing intelligence (SI)	SI1	36.460^***^	0.822	0.777	0.870	0.691
	SI2	38.497^***^	0.820			
	SI3	1.641	Delete			
	SI4	26.718^***^	0.832			
Acting intelligence (AI)	AI1	31.400^***^	0.791	0.739	0.831	0.551
	AI2	21.038^***^	0.717			
	AI3	17.692^***^	0.723			
	AI4	20.171^***^	0737			
Perceived cognitive empowerment (PCE)	PCE1	69.351^***^	0.837	0.816	0.873	0.632
	PCE2	25.510^***^	0.793			
	PCE3	23.279^***^	0.738			
	PCE4	27.526^***^	0.810			
Perceived emotional empowerment (PEE)	PEE1	43.564^***^	0.835	0.801	0.881	0.713
	PEE2	53.695^***^	0.816			
	PEE3	60.456^***^	0.880			
Perceived behavioral empowerment (PBE)	PBE1	42.608^***^	0.907	0.893	0.920	0.744
	PBE2	25.444^***^	0.825			
	PBE3	13.193^***^	0.774			
	PBE4	42.629^***^	0.932			
Automatic features usage intention (UI)	UI1	59.385^***^	0.892	0.857	0.912	0.776
	UI2	41.442^***^	0.855			
	UI3	50.178^***^	0.894			

*** p < 0.001, **p < 0.010, and *p < 0.050.

Next, we evaluated the measurement model for discriminant validity. This involves testing whether the latent variable explains the variance of its indicators better than the variance of other latent variables (Larcker, 1981). Table 4 presents the results of the discriminant validity test, where we compare the square root of AVE values with the correlation between each pair of latent variables. The square root of AVE values on the diagonal for each latent variable is higher than the values of their corresponding correlation coefficients with the other constructs.

**Table 4:** Correlation coefficient matrix and square roots of the AVEs.

	SI	AI	PCE	PEE	PBE	UI
SI	0.831					
AI	0.586	0.742				
PCE	0.532	0.494	0.795			
PEE	0.423	0.388	0.560	0.844		
PBE	0.280	0.151	0.269	0.403	0.863	
UI	0.398	0.392	0.465	0.659	0.203	0.881

*Shaded diagonal elements represent the square roots of the AVEs. SI, sensing intelligence; AI, acting intelligence; PCE, perceived cognitive empowerment; PEE, perceived emotional empowerment; PBE, perceived behavioral empowerment; and UI, automatic features usage intention*.

#### Structural Model Test

Next, we examined the structural model to analyze the significance of the estimated coefficients in the structural part of the model (Hair et al., 1998 2021; Sarstedt et al., 2016). Table 5 summarizes the results of the structural model test. We examined the multiple correlations of parameters representing the relationships between the latent variables, the statistical significance and magnitude of the estimated parameters, and the squared multiple correlations of the structural equations. We used Smart-PLS 3.0 software to test the proposed hypothesis in our study. We used bootstrapping with 5,000 samples to obtain robust results.

**Table 5:** Result of the structural model.

Path Coefficient	Path coefficient	T-statistic	Hypothesis supported(Y/N)
H1a	β_SI - > PCE_ = 0.370	5.349^***^	Y
H1b	β_SI - > PEE_ = 0.298	5.298^***^	Y
H1c	β_SI - > PBE_ = 0.291	5.263^***^	Y
H2a	β_AI - > PCE_ = 0.277	3.445^***^	Y
H2b	β_AI - > PEE_ = 0.213	3.653^***^	Y
H2c	β_AI - > PBE_ = −0.019	0.302n.s.	N
H3a	β_PCE - > UI_ = 0.898	6.339^***^	Y
H3b	β_PEE - > UI_ = 0.542	11.936^***^	Y
H3c	β_PBE - > UI_ = −0.304	6.604^***^	N

*n.s., not significant (two-tailed tests, path comparisons used one-tailed tests). SI, sensing intelligence; AI, acting intelligence; PCE, perceived cognitive empowerment; PEE, perceived emotional empowerment; PBE, perceived behavioral empowerment; and UI, automatic features usage intention*.

^*^p < 0.05;

^**^ *p < 0.01 and*

*** *p < 0.001*.

**Figure 2:**
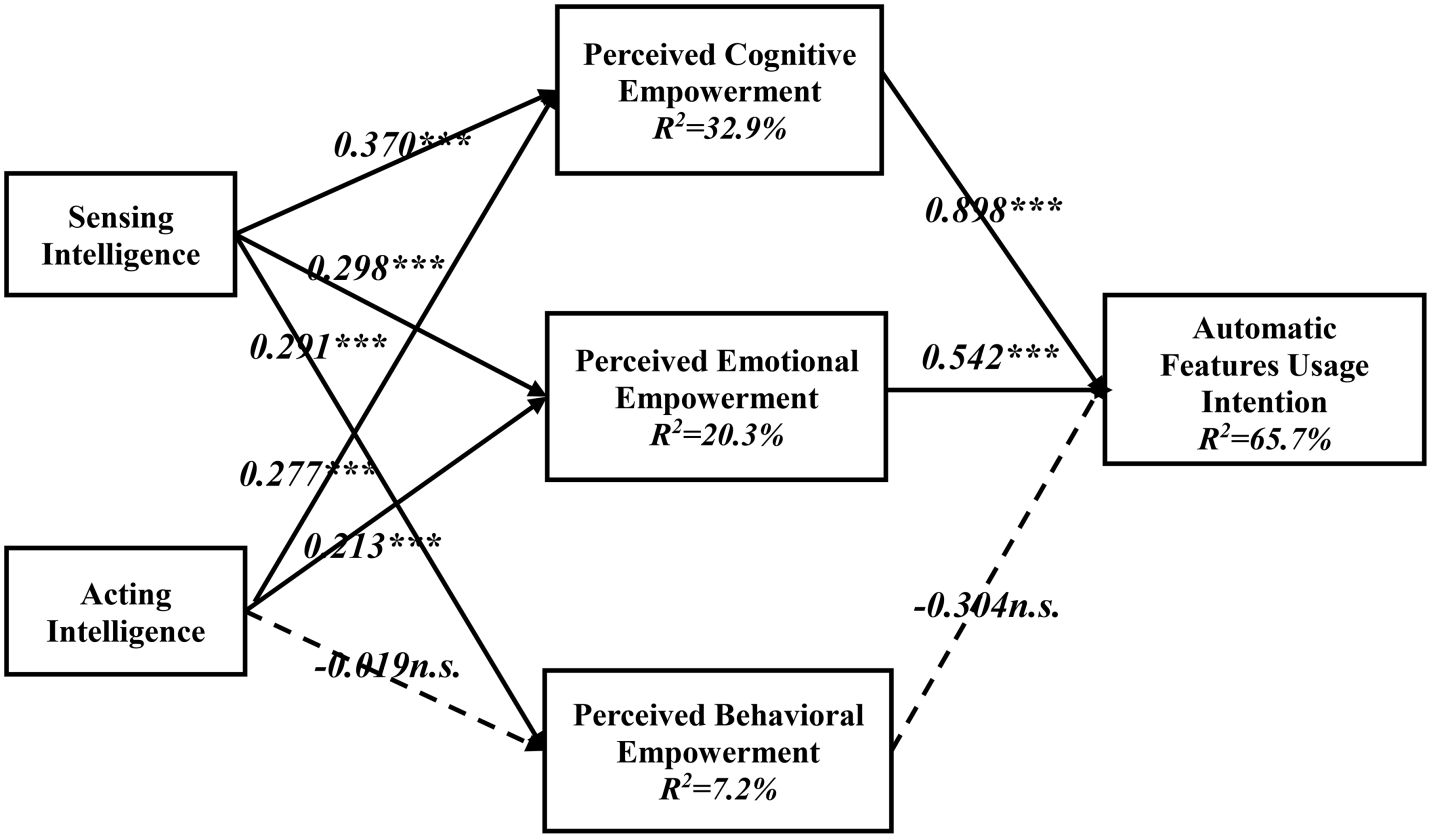
Model testing results (^***^p < 0.01).

Figure 2 summarizes the results of the structural model test. The total variance explained was cognitive empowerment (32.9%), perceived emotional empowerment (20.3%), and perceived behavioral empowerment (7.2%). All the other coefficients were significant at a 0.001 significance level, except the path between acting intelligence, perceived behavioral empowerment, and usage intention. In summary, our results support all hypotheses except H2c and H3c.

#### Mediation and Moderation Tests

We also examined the mediating effect of the three dimensions of perceived empowerment between intelligence and usage intention. We adopted the procedure suggested by Baron and Kenny (1986) to analyze the mediating effect of perceived empowerment. Table 6 presents the results of the mediation test. The results reveal that the perceived empowerment partially mediates the relationship between sensing intelligence or acting intelligence and users’ usage intention. In other words, sensing intelligence and acting intelligence partially influence intention to use automatic features through perceived cognitive empowerment, perceived emotional empowerment, and perceived behavioral empowerment.

**Table 6:** Results of the mediating effect testing.

IV	M	DV	① IV → DV	② IV → M	③ IV + M → DV	
					IV	M
SI	PCE	UI	0.481^***^	0.603^***^	0.282^***^	0.299^***^
SI	PEE	UI	0.481^***^	0.477^***^	0.187^***^	0.579^***^
SI	PBE	UI	0.481^***^	0.360^***^	0.442^***^	0.060
AI	PCE	UI	0.422^***^	0.512^***^	0.230^***^	0.344^***^
AI	PEE	UI	0.422^***^	0.402^***^	0.168^***^	0.592^***^
AI	PBE	UI	0.422^***^	0.374	0.400^***^	0.161^***^

*IV, independent variable; M, mediating variable; DV, dependent variable; SI, sensing intelligence; AI, acting intelligence; PCE, perceived cognitive empowerment; PEE, perceived emotional empowerment; PBE, perceived behavioral empowerment; and UI, automatic features usage intention*.

^*^p < 0.050;

^**^ *p < 0.010 and*

*** *p < 0.001*.

Finally, we tested the moderating effect of the sense of control proposed in this paper on the relationship between perceived empowerment and usage intention. Table 7 presents the results of the test of moderation effect. H4b was supported as expected. However, H4a instead revealed that SoC weakened the positive impact of perceived cognitive empowerment on the usage intention.

**Table 7:** Results of the moderating effect testing.

IV	M	DV	IV × M	① IV → DV	② IV + M → DV		③ IV + M + IV × M → DV		
					IV	M	IV	M	IV × M
PCE	SOC	UI	PCE × SOC	0.150^***^	0.078	0.144^**^	−0.172^*^	0.113^*^	−0.351^***^
PEE	SOC	UI	PEE×SOC	0.585^***^	0.555^***^	0.144^**^	0.392^***^	0.113^*^	0.323^***^
PBE	SOC	UI	PBE × SOC	−0.033	−0.021	0.144^**^	−0.124^*^	0.113^*^	−0.152

*IV, independent variable; M, moderating variable; DV, dependent variable; SI, sensing intelligence; AI, acting intelligence; PCE, perceived cognitive empowerment; PEE, perceived emotional empowerment; PBE, perceived behavioral empowerment; and UI, automatic features usage intention*.

* *p < 0.050*;

** *p < 0.010 and*

*** *p < 0.001*.

In addition, we also tested the effect of SoC on the relationship between sensing and acting intelligence and perceived (cognitive, emotional, and behavioral) empowerment. Table 8 presents the results. We can note that the effect of SI, AI, or SoC on the perceived empowerment alone is much more significant than their interaction effect.

**Table 8:** Results of the moderating effect testing.

IV	M	DV	IV × M	① IV → DV	② IV + M → DV		③ IV + M + IV × M → DV		
					IV	M	IV	M	IV × M
SI	SOC	PCE	SI × SOC	0.542^***^	0.128^***^	0.651^***^	−0.060	0.644^***^	−0.443^***^
SI	SOC	PEE	SI × SOC	0.464^***^	0.207^***^	0.396^***^	0.008	0.376^***^	−0.335
SI	SOC	PBE	SI × SOC	0.313^***^	0.245^***^	0.135	−0.108	0.068	−0.627
AI	SOC	PCE	AI×SOC	0.511^***^	0.058	0.691^***^	−0.066	0.743^***^	−0.291^**^
AI	SOC	PEE	AI×SOC	0.391^***^	0.113^*^	0.444^***^	−0.110	0.463^***^	−0.534^***^
AI	SOC	PBE	AI×SOC	0.197^*^	−0.011	0.338^**^	−0.186^*^	0.153	−0.660

*IV, independent variable; M, moderating variable; DV, dependent variable; SI, sensing intelligence; AI, acting intelligence; PCE, perceived cognitive empowerment; PEE, perceived emotional empowerment; PBE, perceived behavioral empowerment; and UI, automatic features usage intention*.

* *p < 0.050*;

** *p < 0.010 and*

*** *p < 0.001*.

#### Importance-Performance Analysis

To explore how psychological empowerment factors influencing automatic features usage intention further, we also conduct the Importance-Performance Analysis (IPA) of usage intention to automatic features. The results are conducive to improving users’ usage intention to automatic features. It should be noted that the performance analysis here refers to whether the factors can effectively improve users’ usage intention. The Importance-Performance Analysis is an index-based numerical evaluation method that can improve classical regression analysis or path modeling. In other words, this method is used to determine the potential improvement space of variables based on consideration of impact (importance dimension) and predictive index (performance dimension). To be specific, the estimated index (performance dimension) can predict the space for improvement of variables. For example, when the prediction index is high, it indicates that the potential variable has little room for improvement, and thus, there is less possibility that the dependent variable can be further improved. Based on this, we can identify the factors of relative importance and low performance through the Importance-Performance Analysis (Hair et al., 2021). The importance comes from the path coefficient value of PLS path modeling analysis, while the performance is based on the latent variable score, which ranges from 0 to 100. According to the calculation of consumers’ satisfaction index proposed by Anderson and Fornell (2000), the formula for calculating latent variable scores in this paper is shown as follows:


$$\varepsilon_{i}=\left(Ε\left[\varepsilon_{i}\right]-\min\left[\varepsilon_{i}\right]\right)/\left(\max\left[\varepsilon_{i}\right]-\min\left[\varepsilon_{i}\right]\right)*100$$

**Figure 3:**
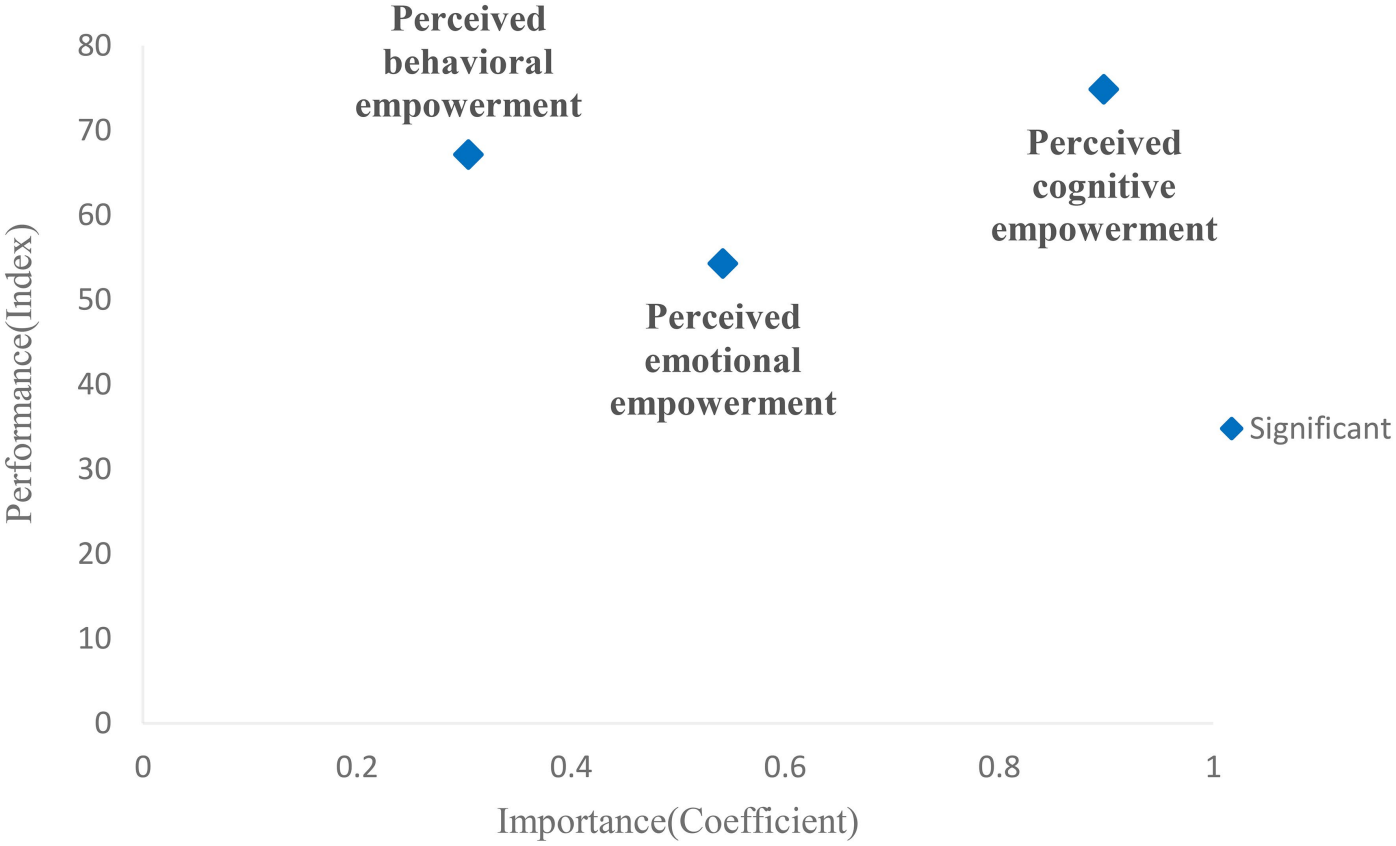
Importance-performance analysis.

It should be noted that ε_i_ is estimated based on the internal path model with non-standardized latent variables. The results are shown in Table 9 below. Based on the results, the Importance-Performance chart is drawn. As shown in Figure 3 below, we take the absolute value of the path coefficient of perceived behavioral empowerment to usage intention to facilitate the comparison since the path coefficient is negative.

**Table 9:** PLS path coefficient and index.

Latent variables	Index	Automatic features usage intention		
		Coefficient	T-value	*p*-value
UI	45.43	-	-	-
PCE	74.89	0.898	6.339	P<0.001
PEE	54.33	0.542	11.9366	P<0.001
PBE	67.16	0.304	6.604	P<0.001

*PCE, perceived cognitive empowerment; PEE, perceived emotional empowerment; PBE, perceived behavioral empowerment; and UI, automatic features usage intention*.

^*^p < 0.050; ^**^p < 0.010 and ^***^p < 0.001.

According to Figure 3, perceived cognitive empowerment is both high importance and high performance compared with emotional and behavioral dimensions. In other words, cognitive empowerment has a significant influence on users’ usage intention, which leaves little room for its performance to improve. The performance index of behavioral empowerment is slightly lower compared to cognitive empowerment, and its effects on usage intention are also much lower than cognitive empowerment. In general, behavioral empowerment is a dimension with relatively low importance and high performance. Finally, the performance of emotional empowerment has a lot of room for improvement as its performance level is the lowest. However, its influence on usage intention is moderate.

## Discussion and Implications

### Discussion of Findings

Based on the empowerment theory, we examined the influence of intelligent vehicles characteristics – sensing intelligence and acting intelligence – on users’ intention to use intelligent features. The results generally support our hypothesis except for H2c and H3c. There could be several reasons for not finding support for these hypotheses. *First*, the direct effect of acting intelligence is significantly greater than the indirect effect through perceived empowerment. We estimate that users may have a weak perception of behavioral empowerment respond to acting intelligence. On the contrary, the direct impact of sensing intelligence is weaker than the mediating effect of perceived empowerment which implies different automatic features may raise users’ intention through different paths. Therefore, implementing differentiated marketing strategies is necessary according to kinds of automatic features. *Second*, compared with the other dimensions of perceived empowerment, the explanatory power of perceived behavioral empowerment is much lower, which may be one of the possible reasons for the insignificance of perceived behavioral empowerment. *Third*, the automation level of current intelligent vehicles is at a low level (that means only automatic features of monitoring, reminding, and warning are enabled). However, the most popular feature in intelligent vehicles is intelligent interaction, which generally empowers drivers in cognitive and emotional dimensions rather than the behavioral dimension. The utilization of features about acting intelligence, such as ACC and APA, is comparatively low. More serious is the fundamental lack of trust that users have in these acting features. Thus, users’ attitudes toward acting intelligence may be a potential barrier to the insignificance of the path coefficient between acting intelligence, perceived behavior empowerment, and usage intention. *Finally*, we first introduced a sense of control into our study as a moderating variable but unexpectedly found that SoC significantly affects perceived behavioral empowerment. Based on this result, we found that other factors may impact perceived behavioral empowerment significantly. This implies that product characteristics, such as acting intelligence, may not be the most important factor for perceived behavioral empowerment.

We also examined the moderating role of the sense of control. Loss of control causes most accidents in intelligent vehicles (Wang et al., 2019). Therefore, a sense of control may increase the safety or trust in intelligent vehicles. However, our results reveal that the relationship between acting intelligence, perceived behavioral empowerment, and usage intention is not significant, thus implying that using automatic features that concentrate on smart interaction is related to cognitive and emotional empowerment. These features have a weak connection to security, and hence a lesser sense of control. The Uncanny Valley theory also contributes to the interpretation. According to this theory, people have favorable emotions toward robots as their humanization increases and negative emotions when they outperform humans. We propose that drivers would attempt to regain control only when the vehicle tends to substitute a drivers’ control over the vehicle. However, this is less likely to happen in L2-level vehicles (Partially automated driving). Consistent with the present intelligent vehicles with a low level of intelligence, users may not be aware of the change in SoC. Thus, as two consequences of empowerment, control, and power have complex relationships, and it is hard to explain their role during the empowerment process based on various contextual factors.

Based on the post-hoc tests, we also found partial mediating effects of perceived empowerment. The results further verified the role of the perceived empowerment mechanism between sensing intelligence, acting intelligence, and usage intention. In summary, in addition to being closely related to perceived cognitive and emotional empowerment, the improvement of usage intention is also inseparable from perceived behavioral empowerment. The automation level of intelligent vehicles will enhance user’s attitudes toward the use of intelligent vehicles. As a result, acting intelligence will also become increasingly valuable over time. Therefore, improving users’ intention to use automatic features from the perspective of perceived behavioral empowerment cannot be ignored.

We conducted the Importance-Performance Analysis (IPA) at the end of the data analysis. In the hypothesis testing, we confirmed the significant impacts of psychological empowerment on usage intention, but it is still not enough for practice. Since we have verified the psychological empowerment affects users’ behavior intention *via* three different dimensions. In contrast, we have not received practical guidance on how to improve users’ intentions in a targeted way yet. Based on this, we also conducted an Importance-Performance Analysis (IPA) on the perceived empowerment of users to improve their usage intention effectively. It was hoped that the analysis could indicate the factors with high importance and low performance, which is of great significance to improve users’ usage intention. To be specific, factors with a low-performance index mean it has a large space for improvement, and factors with high importance mean that it has great importance on the usage intention. In summary, variables with both high importance and low performance can greatly improve users’ intention to automatic features, which expands previous studies that only focused on the influencing factors. Moreover, this analysis also helps us have a deeper understanding of the impact from different dimensions of empowerment on users’ intentions.

As depicted in Figure 3, perceived emotional empowerment is the key variable with relatively high importance and low performance. It indicates that users’ usage intention will be significantly promoted once the perceived emotional empowerment is improved. On the other hand, cognitive empowerment is a factor with high importance and high performance in usage intention, which indicates that cognitive empowerment makes great importance in usage intention and leaves little room for its improvement. This may attribute to the features of intelligent vehicles currently are mostly related to cognitive aspects and thus more easily to be utilized and popularized. As a result, there is little room for the improvement of cognitive empowerment. Finally, the importance index of behavioral empowerment was relatively lower than the other two dimensions, and the room for improvement of performance is also limited. This is probably due to the features provided by intelligent vehicles are monitoring or warning, which are related to cognitive or emotional aspects. Users are not familiar with features related to acting intelligence and have a low acceptance which leads to the importance of perceived behavioral empowerment being much lower than the importance of cognitive and emotional empowerment.

### Theoretical Contributions

This study makes three primary contributions to research by using the novel perspective vof empowerment theory. *First*, with increased advancement in artificial intelligence, it will become a widely used technology in the 21st century. Intelligent driving is at present a hot field of artificial intelligence research, although the amount of research at present is still relatively limited. Manfreda and Ljubi (2021) noted that research on autonomous vehicles is scarce, and the field needs more attention. It is necessary to understand the relevant factors that affect users’ willingness to use. It is also beneficial to assess the future demand and adoption of intelligent vehicles to reduce the resistance toward new technologies (Ismagilova et al., 2019). *Secondly*, the existing literature on empowerment theory explains its two-sided consequences for employees within organizations (Rafiq and Ahmed, 1998). Research, however, has not applied this theory to the context of smart services. Hence, our study extends empowerment theory to the context of intelligent driving. Drawing upon the theory of empowerment, we examined the usage intention of intelligent vehicle technologies, which provides a theoretical basis for future research in intelligent driving. By integrating product characteristics with users’ perceived empowerment, we examined factors influencing the usage intention of intelligent vehicles. *Third*, considering the automatic levels of intelligent vehicles are still at L2 (partially automated driving), previous studies are numerical analyses based on the survey data. However, causal and logical analysis of predictors influencing users’ behavioral intention is lacking. Then, we tried to investigate the influence mechanism of sensing-acting intelligence on users’ usage intention from the perspective of psychological empowerment and revealed the vital role of users’ perceived empowerment in the usage intention of intelligent vehicles. The empirical results show that perceived cognitive empowerment and emotional empowerment have a significant impact on users’ intention to automatic features. This discovery enriches the previous research on intelligent vehicles by verifying the important role of perceived empowerment and provides an empirical basis for us to explore automatic features usage intention. We also confirmed the partial mediating effect of perceived empowerment from the dimensions of perceived cognitive empowerment, perceived emotional empowerment, and perceived behavioral empowerment.

### Practical Implications

As intelligent vehicles are becoming popular, we contribute to this emerging area by investigating automatic features’ usage intention from the empowerment process. This study highlights the key elements which scholars can consider in the future intelligent vehicles industry. *First*, sensing intelligence and acting intelligence are both critical to users, improving users’ perceived empowerment. Sensing contributes more to perceived empowerment than acting, noting that sensing intelligence in vehicles should be given more attention to designing intelligent vehicles. Still, we cannot neglect the acting intelligence which results from sensing. Acting also, to some extent, enhances users’ intention toward automatic features through perceived cognitive and emotional empowerment. Overall, this study shows that it is feasible to improve the empowerment of users by emphasizing the intelligence of intelligent vehicles to enhance their usage intention. *Secondly*, we confirm that perceived empowerment in intelligent vehicles contributes to increased intention for using automatic features. Specifically, perceived cognitive empowerment contributes more to users’ intentions than perceived emotional and behavioral empowerment. This may provide some clues for designing or marketing the latest models of intelligent vehicles. Intuitively speaking, improving perceived cognitive empowerment and emotional empowerment of users is essential for using automatic features. *Third*, a sense of control is an important outcome of the empowerment process. Control over vehicles is essential, especially in intelligent driving, as previous accidents attributed to the loss of control. Thus, SoC also helps ensure security and fosters users’ trust in intelligent vehicles (Sharan and Romano, 2020). We can conclude that SoC facilitates users’ perceived empowerment and behavioral intentions than its interaction effects with empowerment or intelligence. In other words, results of the moderating effect indicate that SoC will directly increase the perception of empowerment and people’s usage intention.

### Limitations

We should interpret the results of this study considering its limitations. *First*, our sample was from China. Hence, the generalizability of the results is limited to similar contexts. We can also collect data from other countries to check whether there are cultural differences in the results of our study. *Second*, based on the rapid development of intelligent vehicles, we collected data at a single point in time, limiting our observations for further insight into the use of intelligent vehicles. For example, we can include longitudinal studies to capture the changes in the use of intelligent vehicles. In the future, researchers can conduct experimental research to expand existing literature and theories. *Third*, regardless of intelligent vehicles’ sensing and acting intelligence, other characteristics, such as anthropomorphism also deserve more attention and investigation. Similarly, we neglected various factors, such as trust or perceived value, to focus on users’ perceived empowerment in the intelligent driving scenario. Future studies may consider more factors and theories to enrich the research in the intelligent driving field. *Finally*, the automatic level of intelligent vehicles currently is L2 which leaves us with a possibility to explore the adoption of higher levels of intelligent vehicles.

## Conclusion

Our study examined how sensing and acting intelligence of intelligent vehicles affect drivers’ intention to use them from the empowerment theory. Our results show that sensing and acting intelligence contribute to perceived empowerment and behavioral intention of users. In addition, the influence on cognitive empowerment is much greater than emotional and behavioral empowerment. As a result, cognitive empowerment is a crucial factor that enhances users’ intention to use automatic features. Our research thus increases the scope of application and interpretation of the empowerment theory, and it has also increased the understanding of users’ perceived empowerment followed by their usage intention. The results provide a reference for the design and marketing of intelligent vehicles, enhance users’ perceived empowerment, and improve users’ behavioral intentions toward intelligent vehicles currently and in the future.

## Data Availability Statement

The original contributions presented in the study are included in the article/supplementary material, and further inquiries can be directed to the corresponding author.

## Ethics Statement

Ethical review and approval were not required for the study on human participants in accordance with the local legislation and institutional requirements. Written informed consent for participation was not required for this study in accordance with the national legislation and the institutional requirements.

## Author Contributions

TL: conceptualization, formal analysis, and writing – original draft. SG and HZ: review and editing. All authors contributed to the article and approved the submitted version.

## Funding

This work was supported by the grants from the National Natural Science Foundation of China (project no. 71810107003) and the National Social Science Fund of China (project no. 18ZDA109).

## Conflict of Interest

The authors declare that the research was conducted in the absence of any commercial or financial relationships that could be construed as a potential conflict of interest.

## Publisher’s Note

All claims expressed in this article are solely those of the authors and do not necessarily represent those of their affiliated organizations, or those of the publisher, the editors and the reviewers. Any product that may be evaluated in this article, or claim that may be made by its manufacturer, is not guaranteed or endorsed by the publisher.

## Figures and Tables

**Table:** 

Construct	Operational definition	Subdimensions	Reference
Sensing Intelligence	The extent to which Intelligent vehicles can monitor the surroundings and sense the information	Intelligent vehicles can be aware of the state of their surroundings. Intelligent vehicles can recognize information from the environment. Intelligent vehicles can recognize objects in the environment. Intelligent vehicles can monitor the status of objects in the environment.	Hu et al., 2021
Acting Intelligence	The extent to which Intelligent vehicles can make safety responses based on the perceived information	Intelligent vehicles can complete the operation of the skill driving. Intelligent vehicles can implement the operation of driving. Intelligent vehicles can perform the operation of driving. Intelligent vehicles can carry out the operation of driving.	Hu et al., 2021
Cognitive Empowerment	The extent to which intelligent vehicles strengthen the cognition of the surroundings and improve understanding of the vehicle’s function or use.	Intelligent vehicles are conscious of the driving environment. Intelligent vehicles can help Distinguish different conditions. Intelligent vehicles are aware of the environment. Intelligent vehicles can Understand what happens.	Christens, 2012; Aleksander, 2017
Emotional Empowerment	The extent to which intelligent vehicles enhance confidence and positive emotions in driving.	Intelligent vehicles increase perceived control when I was driving. Intelligent vehicles increase Self-efficacy when I was driving. Intelligent vehicles increase perceived competence when I was driving.	Speer and Peterson, 2000; Christens, 2012
Behavioral Empowerment	The extent to which intelligent vehicles enhance operating skills and activities of driving.	Intelligent vehicles enhanced professional Skills of driving. Intelligent vehicles enhanced Involvement/Participation in driving. Intelligent vehicles enhanced the Coping behaviors of driving.	Speer and Peterson, 2000; Christens, 2012
Sense of control	The extent to which users believe their driving actions can change the driving outcome.	I have a great deal of control over how to drive intelligent vehicles. I have freedom in determining how to drive intelligent vehicles. I have a chance to use personal initiative on driving intelligent vehicles. I have decision-making opportunities while driving intelligent vehicles.	Kim and George, 2005; Li and Gregor, 2011
Automatic features usage intention	The extent of users’ is willing to use the automatic features.	I predict that I will use the automatic features when they become available. I intend to use the automatic features when they become available. I plan to use the automatic features when they become available.	Komiak and Benbasat, 2006; Qiu and Benbasat, 2008

## Footnotes

^1^ https://www.wjx.cn/
